# Characterization of rye flours and their potential as reference material for gluten analysis

**DOI:** 10.1016/j.foodchem.2022.135148

**Published:** 2023-05-15

**Authors:** Majlinda Xhaferaj, Gabriella Muskovics, Eszter Schall, Zsuzsanna Bugyi, Sándor Tömösközi, Katharina A. Scherf

**Affiliations:** a Karlsruhe Institute of Technology, Institute of Applied Biosciences, Department of Bioactive and Functional Food Chemistry, Karlsruhe, Germany; b Budapest University of Technology and Economics, Department of Applied Biotechnology and Food Science, Research Group of Cereal Science and Food Quality, Budapest, Hungary

**Keywords:** Celiac disease, Enzyme-linked immunosorbent assay (ELISA), Polyacrylamide gel electrophoresis (SDS-PAGE), Reversed-phase high-performance liquid chromatography (HPLC), Secalins, Wheat allergy

## Abstract

The safety of gluten-free products relies on accurate gluten analysis, most commonly using ELISA. These test kits are calibrated to gliadins or wheat gluten, because there is no reference material (RM) for rye. Our aim was to select representative samples out of 32 rye cultivars for use as RM. All cultivars were characterized by RP-HPLC, gel permeation HPLC and R5 and G12 ELISA. The protein and gluten content ranged from 5.5 to 11.2 g/100 g and 3.0 to 7.8 g/100 g, respectively. The average protein distribution was 40% albumins/globulins, 23% γ-75k-secalins, 17% γ-40k-secalins, 14% ω-secalins and 6% high-molecular-weight-secalins. The mean prolamin/glutelin ratio was 4.4 for rye and this translates to an estimated conversion factor from rye prolamins to gluten of 1.2, instead of the usual factor of 2. Seven cultivars were selected for RM production based on cluster analysis, geographical origin and availability to comprehensively cover the diversity of rye.

## Highlights

• Comprehensive protein characterization of 32 different international rye cultivars.
• Selection of seven cultivars for the production of a new gluten reference material.
• The prolamin/glutelin ratio as 4.4 for rye, and not 1, as often assumed.
• The common division into prolamins and glutelins is not suitable for rye secalins.

## Graphical abstract

**Figure:**
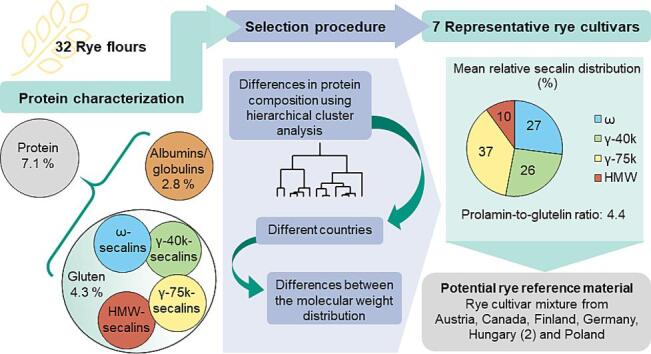

## 1 Introduction

Gluten is a complex mixture of storage proteins present in the starchy endosperm of grains such as wheat, rye, and barley. Rye gluten contains the following protein types: high-molecular-weight (HMW)-, ω-, γ-75k- and γ-40k-secalins. Based on homologous amino acid sequences to wheat gluten proteins, ω- and γ-40k-secalins can be defined as prolamins and HMW-, γ-75k- secalins as glutelins. Gluten is of considerable concern, because it may cause hypersensitivity reactions such as celiac disease (CD), non-celiac gluten sensitivity and wheat allergy in predisposed individuals. Its consumption triggers inflammation of the upper small intestine with infiltration of intraepithelial lymphocytes and partial to total villous atrophy in CD patients. Because a lifelong gluten-free diet is the only treatment that shows an improvement of gastrointestinal symptoms, CD patients depend on the correct analysis of gluten from foods (Choung et al. 2017).

The declaration “gluten-free” and the analysis of gluten from foods are precisely regulated. According to the Codex Alimentarius, gluten-free labelled foods must not exceed 20 mg of gluten per kg product. The most common methods to quantitate gluten in foods are ELISA test kits. More specifically, the R5-Méndez method has been recognized as a type I method for gluten analysis (Lacorn et al. 2019). Other ELISA assays such as G12 are also acceptable with similar performance parameters. The ELISA uses antibodies to target specific epitopes within the gluten proteins. The R5 mAb, for example, was raised against secalins and primarily recognizes the epitope with the amino acid sequence QQPFP. This epitope is repetitively present in many peptides derived from gliadins (wheat prolamins), secalins, and hordeins (barley gluten) that are toxic or immunogenic for CD patients. However, different ELISA test kits do not always achieve the same result, due to differences in extraction methods, reference materials (RM) used for calibration and specificity of antibodies (Amnuaycheewa et al. 2022; Panda et al. 2017).

Prior to the analysis, gluten must be extracted depending on the food matrix (Amnuaycheewa et al. 2022). The Osborne fractionation is commonly used to extract gluten, where first the water- and salt-soluble albumins and globulins are extracted to access the prolamins, which are extractable using 60% ethanol. Finally, the glutelins, which are disulfide-linked proteins, are extracted using alkaline- or acid-based agents (Osborne, 1895). The extraction procedure for ELISA tests differs depending on the manufacturer. Various buffers are available containing, for example, β-mercaptoethanol and phosphate-buffered saline or 60% ethanol. Moreover, most ELISA antibodies primarily target the prolamin fraction. The gluten content is then calculated with a conversion factor of two, because the prolamin content is typically assumed to be 50% of the gluten content. The validity of this conversion factor is questioned, because the prolamin and glutelin content varies strongly depending on cereal species, cultivar and processing from raw material to final product (Wieser and Koehler 2009).

The RM used in the ELISA is composed of wheat prolamins. The so-called Prolamin Working Group (PWG)-gliadin RM is successfully used as a calibrator in ELISAs, because it is the best characterised RM available for gluten analysis. It was isolated from a mix of 28 different European wheat cultivars (van Eckert et al. 2006). Different types of RM have been proposed for gluten quantitation including recombinant proteins, flours or isolated gluten protein types (Huang et al. 2017; Schalk et al. 2017). Due to the considerable variability in gluten composition, gluten RM development encounters several difficulties. The gluten composition of cereals is influenced by environmental and genetic factors which cannot be eliminated (Hajas et al. 2017; Schall et al. 2020; Uhlen et al. 2015). Recent RM developments showed that mixing different cultivars (flours) significantly reduced the effect of genetic and environmental variability (Schall et al. 2020). Most studies focus on wheat gluten composition for use as a RM (Bugyi et al. 2012; Hajas et al. 2018; Schall et al. 2020; van Eckert et al. 2006), whereas little research has been done on other cereal proteins such as secalins from rye (Gellrich et al., 2003, Schalk et al., 2017).

The differences in fully characterized gluten of wheat, rye and barley flours were shown by Schalk et al. They reported a less clear separation of rye prolamins and glutelins according to their solubility (Osborne fractionation) compared to wheat. γ-75k-secalins and a minor part of HMW-secalins appeared in both fractions (Lexhaller et al. 2019; Schalk et al. 2017). The classification of rye fractions into prolamins and glutelins must therefore be viewed critically. To date, there is no rye RM available for gluten quantitation. In addition, the protein composition of a larger collection of different international rye cultivars has never been reported. To fill this gap, the aim of this study was to select specific rye cultivars with a potential as a new RM for ELISA and other analytical methods. Based on previous work on RM from wheat (Hajas et al. 2018; Schall et al. 2020), the hypothesis is that a mixture of different rye cultivars is more suitable compared to a single cultivar to reduce genetic and environmental variability of gluten composition and to be as representative as possible for the various cultivars grown around the world. The leading countries in rye production are the European Union (7.9 million tons), Russia (1.4 million tons), Belarus (0.6 million tons), Ukraine (0.3 million tons) and Canada (0.3 million tons). The novelty of the work lies in the comprehensive characterization of 32 rye cultivars from various countries (mostly from the European Union) and the selection of seven suitable cultivars representing the variability of rye protein composition. A blend of these seven cultivars will be used as a basis for the new RM. Further, accurate prolamin/glutelin ratios are reported for rye for the first time, allowing the calculation of a rye-specific factor to convert the prolamin content analysed by ELISA to gluten.

## 2 Materials and methods

### 2.1 Sourcing of rye grains and flour preparation

Rye samples (n = 32) were collected from eight different geographical origins (Table S1). Kernels were milled on a laboratory mill (Cyclotec Mill 1093, Foss Tecator AB, Höganäs, Sweden) to wholemeal flours. The mill was cleaned mechanically and with compressed air after each sample and the first 10 g of newly milled sample were discarded. Wholemeal flours were stored in zip-lock bags at 22 °C until further use. For the rye flour mixture of the seven selected cultivars (Table 1), 500 mg of flour each were mixed and homogenized for 24 h in an overhead shaker.

### 2.2 Rye flour characterization

The crude protein content of the flours was determined by the Dumas method using a Leco FP 528 nitrogen analyser (Leco Corporation, St. Joseph, USA) in duplicates following ICC Standard No. 167. The nitrogen content was multiplied by 5.7 to obtain the crude protein content. Crude fat content measurement of the flours was performed in duplicates according to ICC Standard No. 136 using a Soxtec System HT-1043 instrument (Foss Tecator AB, Höganäs, Sweden). The moisture content of the flours was determined by the oven-drying method in duplicates according to ICC Standard No. 109/1.

### 2.3 Rye protein characterization

#### 2.3.1 Extraction procedure

The extraction of proteins from rye flours was performed according to the modified Osborne fractionation procedure (Wieser et al. 1998). Flour (100 mg) was extracted stepwise with the following extraction solutions: saline (SSol) (400 mmol/l NaCl + 67 mmol/l Na_2_PO_4_/KH_2_PO_4_ (pH 7.6)), 60% aqueous ethanol (ESol), and buffer solution (BSol) (50% (v/v) 2-propanol + 50 mmol/l TRIS HCl (pH 8.0) + 12 g urea + 10 mg/ml dithiothreitol (DTT)). First, albumins and globulins (ALGL) were extracted twice with 1 ml of SSol, followed by vortexing for 2 min, magnetic stirring for 10 min, and centrifugation for 25 min at 25 °C at 3550 rcf. Second, prolamins were recovered by triplicate extraction with 0.5 ml of ESol, followed by the steps described for ALGL. Part of the prolamin fraction was additionally reduced with 1% (w/v) DTT. To obtain glutelins, extraction was performed twice with 1 ml BSol under nitrogen, followed by vortexing for 2 min, magnetic stirring for 30 min at 60 °C in a water bath, and centrifugation for 30 min at 25 °C and 3550 rcf. The extracts of ALGL, prolamins, reduced (red.) prolamins and glutelins were diluted to 2 ml with the respective extraction solvents, filtered (0.45 µm Whatman SPARTAN, Cytiva Europe GmbH, Freiburg im Breisgau, Germany) and used for HPLC analysis of the proteins.

#### 2.3.2 Protein characterization by RP-HPLC

**Fig. 1:**
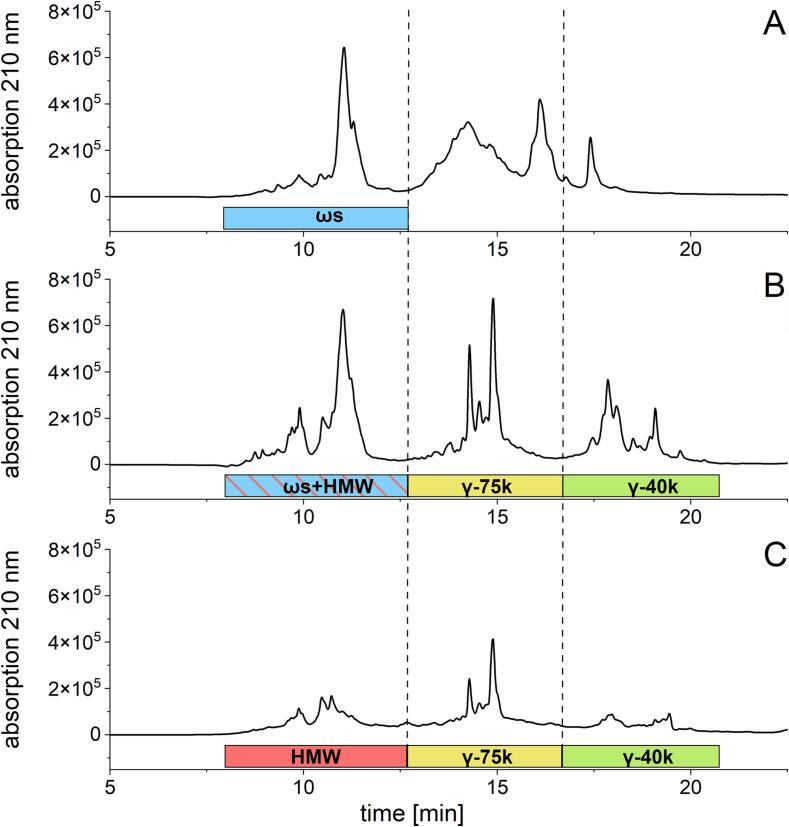
**RP-HPLC profiles of the Osborne fractions of the rye cultivar Elias (ELI_AUS20)**. A: Unreduced prolamins, B: Reduced prolamins, C: Glutelins. With the rye protein fractions ωs: ω-secalins; HMW: high-molecular-weight secalins; γ-75k: γ-75k-secalins and γ-40k: γ-40k-secalins.

Protein separation was carried out on a Shimadzu Prominence HPLC (Shimadzu, Nakagyo-ku, Kyoto, Japan) using the following stationary phase: YMC Triart Bio C_18_, 150 mm × 2.1 mm, 3 µm (YMC Europe GmbH, Dinslaken, Germany). The mobile phase consisted of A: 0.1% trifluoroacetic acid (TFA) in bidist. water and B: 0.1% TFA in acetonitrile. For separation of the ALGL fraction, a flow rate of 0.5 ml/min was used with gradient elution: 0–0.4 min, 0% B; 0.5 min, 20% B; 8 min, 60% B; 8.1 min, 100% B; 8.1–13 min, 100% B; 13.1–27 min 0% B. The gradient elution of prolamins and glutelins with a flow rate of 0.5 ml/min was done as follows: 0–0.4 min, 5% B; 0.5 min, 30% B; 16 min, 60% B; 16.1–22.1 min, 100% B; 22.2 min, 5% B; 22.2–35 min, 0% B. Both gradients were operated at a column temperature of 60 °C. Proteins were detected at 210 nm and quantitated using the corresponding absorbance areas of PWG-gliadin (van Eckert et al. 2006). The gluten content was calculated from the sum of red. prolamins and glutelins. The protein types were quantitated based on their percentage of the total peak area. The evaluation of the chromatographic profiles and the classification of the protein fractions was based on the literature (Gellrich et al., 2003, Schalk et al., 2017) (Fig. 1).

#### 2.3.3 Relative molecular mass distribution by GP-HPLC

The relative molecular mass (M_r_) distribution of gluten proteins was determined using gel permeation (GP)-HPLC. GP-HPLC analysis was performed using a Shimadzu Nexera XS HPLC (Shimadzu, Nakagyo-ku, Kyoto, Japan). The column was a BioSep-SEC-s3000 (300 × 4.6 mm, 5 µm, Phenomenex, Aschaffenburg, Germany) operated at 22 °C. The mobile phase consisted of A: 0.1% TFA in bidist. water and B: 0.1% TFA in acetonitrile. The flow rate was 0.3 ml/min with an isocratic elution (50% A, 50% B). Detection was performed using a DAD at 210 nm. Proteins with known M_r_ were used to determine the integration limits for specific M_r_ ranges. The proteins used were cytochrome *C* from horse heart (12.4 kDa), carbonic anhydrase from bovine erythrocytes (29 kDa) and albumin from bovine serum (66 kDa). The M_r_ ranges were the following: (1) > 66 kDa; (2) 66–29 kDa; (3) 29–12.4 kDa; and (4) < 12.4 kDa. In each range, the area under the curve (AUC) was integrated and calculated as a percentage of the total area.

#### 2.3.4 SDS-PAGE

Rye flour (20 mg) of the selected samples was extracted with 1 ml of the extraction buffer (293.3 mmol/L sucrose, 246.4 mmol/L TRIS, 69.4 mmol/L SDS, 0.51 mmol/L EDTA, 0.22 mmol/L Brilliant Blue G-250, 0.177 mmol/L phenol red, 0.105 mmol/L HCl, pH 8.5) overnight under reducing conditions (DTT, 50 mmol/L). The flour suspension was then shaken for 10 min at 60 °C and centrifuged at 2370 rcf for 5 min at 20 °C. The supernatant (10 µL) was used for the electrophoresis. A NuPAGE 4–12% BIS-TRIS protein gradient gel (1.0 mm, 10-well, Invitrogen, Carlsbad, CA, USA) and MOPS running buffer (50 mmol/L MOPS, 50 mmol/L TRIS, 3.5 mmol/L SDS, 1 mmol/L EDTA, pH 7.7) were used. Prior to use, DTT (5 mmol/L) was added to the buffer as reducing agent (Lagrain et al., 2012, Geisslitz et al., 2020). The samples and the marker (5 µL) (PageRuler Unstained Protein Ladder (Thermo Scientific, Bremen, Germany) covering a range of 10 to 200 kDa with 14 proteins) were loaded into the wells. The gels were run at 200 V and 115 mA according to the manufacturer’s guidelines (Thermo Scientific) with a running time of 30 min. Then the proteins were fixed for 30 min in 12% trichloroacetic acid and stained with Coomassie Brilliant Blue G-250 (3 mmol/L) in water/methanol/acetic acid (454/454/92, v/v/v) for 30 min. The gels were first destained twice with methanol/water/acetic acid (50/40/10, v/v/v) for 15 min and with water/methanol/acetic acid (80/10/10, v/v/v) until the bands were visible (Kasarda et al. 1998). The gels were scanned with a Gel Doc EZ Imager (BioRad, Feldkirchen, Germany) and the M_r_ of the bands were estimated based on the marker proteins by the AIDA Image Analysis software.

### 2.4 Gluten quantitation by ELISA

Gluten quantitation was performed with two commercially available ELISA test kits: RIDASCREEN Gliadin Assay (limit of detection (LOD): 0.5 mg/kg of gliadin, limit of quantitation (LOQ): 2.5 mg/kg) (R7001, R-Biopharm, Darmstadt, Germany) and AgraQuant Gluten G12 Assay (LOD: 2.0 mg/kg of gluten, LOQ: 4.0 mg/kg) (COKAL0200, Romer Labs, Tulln, Austria). These two kits apply different antibodies (R5 mAb and G12 mAb, respectively) and different calibrators (PWG-gliadin and wheat gluten extract, respectively). ELISA procedures were carried out according to the kit instructions. Rye flour extracts were additionally diluted 10.000-fold to obtain a sample concentration in the calibration range. The absorbances were determined using a microplate reader (iMarkTM Microplate Absorbance Reader, Bio-Rad, Hercules, CA, USA). The gluten concentrations were calculated from the absorbance values by the Bio-Rad Microplate Manager 6 software (Bio-Rad) using the curve fit and calculations suggested by the test kit manufacturer, respectively. The gluten content is calculated using the conversion factor of 2 (gliadin content × 2) for the RIDASCREEN Gliadin Assay.

### 2.5 Statistics

Mean values (n = 3) and absolute standard deviations (SD) were calculated for all quantitative values of each rye cultivar. The Pearson correlation coefficients (r) were defined as r ≤ ± 0.54 no correlation, ± 0.54 < r ≤ ± 0.67 weak correlation, ± 0.67 < r ≤ ± 0.78 medium correlation, and r > ± 0.78 strong correlation (Thanhaeuser et al. 2014). Additionally, a one-way ANOVA, (Tukey’s post hoc test, p < 0.05) was used to determine significant differences among the means of the M_r_ distribution (GP-HPLC) and all parameters of protein composition. To find similarities and differences among rye cultivars, a hierarchical cluster analysis was performed. All statistical analyses were carried out using Origin 2021b software (OriginLab Cooperation, Northampton, MA, USA).

## 3 Results and discussion

### 3.1 Moisture, fat and protein content

**Fig. 2:**
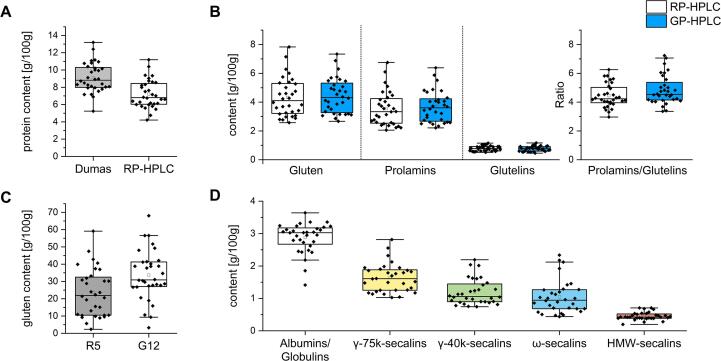
**Boxplots showing the protein characterization of 32 rye cultivars.** The box represents the 25th and 75th percentiles. The diamonds represent the data points for each cultivar (n = 32). The small square in the box indicates the mean, the line the median and the whiskers indicate the upper (75th percentile) and lower (25th percentile) inner fence with a 1.5 interquartile range (whisker length determined by the outermost data point that falls within upper and lower inner fence). A: Comparison of the protein content measured with Dumas and RP-HPLC. B: Gluten, prolamins, glutelins and the prolamin-to-glutelin ratio measured with RP-HPLC and GP-HPLC. C: Gluten content measured with R5 and G12 ELISA in comparison. D: Protein fractions measured with RP-HPLC.

The moisture content of the 32 rye samples ranged from 8.2 to 13.0% with a mean moisture of 10.4 ± 1.0%. The samples had an average fat content of 1.2 ± 0.4% (Tables S2 and S3). The results of the in-depth characterization of 32 rye cultivars are presented in Tables S3-S5 and Fig. 2. The protein content of the samples ranged from 5.2 to 13.2% by Dumas and from 4.2 to 11.2% by RP-HPLC. In general, the protein content by Dumas was on average 1.9 g/100 g higher compared to the sum of protein fractions (albumins, globulins, red. prolamins and glutelins) measured by RP-HPLC (Fig. 2A) and there was a positive correlation (r = 0.98) between the results of both methods. The difference in the protein content comparing both methods is mainly due the presence of unextractable proteins in the residue after the third extraction step. Additionally, the crude protein content is subsequently calculated using a species-specific conversion factor to calculate from nitrogen to protein. However, food can also contain non-protein nitrogen such as nitrate, ammonia, urea, nucleic acids or free amino acids. This can be an additional reason why the crude protein content is higher compared to the RP-HPLC method, as has been reported before (Schalk et al. 2017). Previous studies evaluating the crude protein content of different rye samples showed similar results to ours ranging from 5.1 to 13.6% of total protein (Gellrich et al., 2003, Schalk et al., 2018). More recently, the protein content of five rye cultivars ranged from 8.0 to 11.4% using the Kjeldahl method (Rani et al. 2021). The differences in content are, apart from the method of determination, strongly influenced by several factors such as the genetic variability and environmental factors such as the harvest year, fertilization and country of origin (Fowler et al. 1990). Accordingly, differences in gluten content and composition are expected as well.

### 3.2 Gluten quantitation using RP-HPLC

The gluten content obtained by RP-HPLC ranged from 2.6 to 7.8 g/100 g of flour (Table S2). These results agree with previous reports where the gluten content of rye flour is usually lower compared to wheat flour (Schalk et al. 2017). The content of protein fractions ranged from 1.4−3.6 g/100 g for ALGL, 1.0–2.8 g/100 g for γ-75k-secalins, 0.7–2.2 g/100 g for γ-40k-secalins, 0.4–2.3 g/100 g for ω-secalins and 0.2–0.7 g/100 g for HMW-secalins (Fig. 2D, Tables S2 and S4). Considering the mean values of the protein fractions of the 32 rye cultivars, the relative protein distribution was 40% ALGL, 23% γ-75k-secalins, 17% γ-40k-secalins, 14% ω-secalins and 6% HMW-secalins. Few studies have investigated the protein distribution of the rye protein fractions (Gellrich et al., 2003, Schalk et al., 2017, Rani et al., 2021). However, the distribution pattern of rye gluten fractions shown (γ-75k-secalins > γ-40k-secalins > ω-secalins > HMW-secalins) was confirmed by our results for most of the samples (Fig. 2D).

### 3.3 Gluten quantitation and relative molecular mass distribution using GP-HPLC

The gluten content analyzed by GP-HPLC ranged from 2.7 to 7.3 g/100 g of flour (Table S2). As expected, the results obtained by GP-HPLC were very similar to those by RP-HPLC and there was a strong positive correlation (r = 0.98). To determine the M_r_ distribution, the chromatograms were subdivided into four ranges: (1) > 66 kDa; (2) 66–29 kDa; (3) 29–12.4 kDa; and (4) < 12.4 kDa (Table S5). Considering the mean values, the prolamins showed a distribution of 29.6% (1), 10.6% (2), 25.1% (3) and 34.6% (4). Within the prolamins, the sample RET_FIN20 stood out significantly with lower percentages of 19.9% (1) and 15.6% (3) and the highest percentage of (4) with 54.2%. After reduction of the prolamins, the average distribution changed to 5.7% (1), 11.3% (2), 52.4% (3) and 30.6% (4). The higher M_r_ fraction (1) decreased and fraction (3) increased according to expectations due to the reduction of the disulfide bonds of the proteins using DTT. Within the red. prolamins, the greatest difference between cultivars was observed in fraction (2), which ranged from 6.8% (DAC_CAN17) to 22.1% (DR_HUN19). The distribution of fraction (2) was low for all the Canadian samples and raged from 6.8 to 7.7% (Table S5). The distribution in the glutelin fraction was comparably homogenous within the samples and the M_r_ categories and resulted in the following average distribution: 12.6% (1), 12.7% (2), 33.5% (3) and 41.3% (4). The differences in the M_r_ distribution were used as an additional selection criterion (see 3.6).

### 3.4 Gluten quantitation with ELISA

The commonly used R5 sandwich ELISA according to Méndez and the G12 sandwich ELISA were used for gluten quantitation (Méndez et al., 2005, Morón et al., 2008). For most samples, the gluten content was overestimated in both kits compared to RP-HPLC results (Table S3), except for WIB_HUN17 (ELISA recovery of 76.9% compared to RP-HPLC) using the R5 ELISA and DA_HUN17 (recovery of 96.1%) using the G12 ELISA. The gluten content measured by G12 ELISA was on average 26.7% higher compared to the results obtained using R5 ELISA. Regarding the gluten content, there was a strong correlation (r = 0.82) between the results of both ELISA kits. Comparing both against the RP-HPLC results, a medium correlation was found (R5, r = 0.76; G12, r = 0.71). The results show that different ELISA methods do not always achieve the same result, due to different specificities of the antibodies, as has been reported before (Scherf, 2017, Yu et al., 2021, Amnuaycheewa et al., 2022).

### 3.5 Conversion factor for rye gluten content estimation

The prolamin/glutelin ratio is relevant for the calculation of the gluten content, because the R5 and G12 ELISA mainly target the alcohol-soluble prolamin fraction. According to the Codex, gluten is calculated by duplication of the prolamin content, based on the assumption that the prolamin/glutelin ratio is 1. However, this is often not the case (Wieser and Koehler 2009). Ultimately, this leads to an under- or overestimation of gluten. Furthermore, the R5 ELISA kit uses the PWG-gliadin standard for calibration. Since wheat prolamins differ from rye prolamins, a suitable rye-based RM is needed for more appropriate calibration. Our findings confirm the overestimation of rye gluten (Lacorn et al., 2019), because a higher content was found using both ELISA kits compared to RP-HPLC (Fig. 2C, Tables S2 to S4). The main reason for the overestimation is the higher sensitivity of the R5 and G12 antibodies against rye proteins compared to wheat proteins (Lexhaller et al. 2016).

The prolamin/glutelin ratio for the 32 rye flours ranged from 3.0 to 6.3, with an average of 4.4 (Table S2). The ratio for rye is thus considerably higher compared to the 1.4 to 3.6 reported for wheat (Pronin et al., 2020). This difference can be explained by the presence of γ-40k- and γ-75k-secalins in both prolamin and glutelin fractions, as can also be observed in the RP-HPLC chromatograms (Fig. 1). Therefore, the clear distinction of rye proteins, based only on the solubility of the Osborne fractionation (prolamins and glutelins) is less applicable for secalins, resulting in high prolamin/glutelin ratios and overestimation of gluten in ELISA test kits when calibrated to gliadins or wheat gluten. Based on the average prolamin/glutelin ratio of 4.4 (±0.8), the conversion factor from rye prolamins to gluten is estimated to be 1.2, instead of 2. Using this rye-specific factor will already reduce overestimation of rye gluten. Using the conversion factor of 1.2 on the mean ELISA values for R5 (23.1 g/100 g of gluten) and G12 (32.8 g/100 g of gluten), the mean values are lower for both ELISA kits (R5: 13.9 g/100 g of gluten; G12: 19.7 g/100 g of gluten). However, the values are still higher compared to RP-HPLC, because the conversion factor alone does not fully correct the high reactivity of the R5 and G12 antibodies to rye gluten.

### 3.6 Rye cultivar selection for the reference material

The production of the potential RM requires a selection of representative rye cultivars from the whole collection of 32 samples based on qualitative and quantitative criteria. The first criterion was a typical RP-HPLC (Fig. 1) and GP-HPLC elution profile. Visual examination and comparison of the individual profiles revealed slight differences between the samples. Overall, the RP-HPLC elution profiles of all 32 flour samples investigated were comparable to the profiles from previous studies (Gellrich et al., 2003, Gessendorfer et al., 2009, Schalk et al., 2017). The same was observed for the GP-HPLC elution profiles. Thus, all of the 32 samples were taken into account for further investigations.

The quantitative criteria focused on similarities and differences in cultivar characteristics to cover the variability in protein composition. For this purpose, cluster analysis was carried out on all quantitative data (Tables S3 to S5). The aim was to create clusters with samples showing similar characteristics and select samples from different clusters to cover the variability of rye. The hierarchical cluster analysis resulted in the formation of 5 clusters (Table S1). The first cluster (C1) contained 14 cultivars from different countries including Canada (7), Germany (2), Estonia (2), Latvia (1), Poland (1) and Hungary (1). The second cluster (C2) contained 4 cultivars from Hungary only. Cluster 3 (C3) contained 7 cultivars (Hungary (2), Poland (2), Austria (2), Estonia (1)). Cluster 4 (C4) contained 6 cultivars (Hungary (4), Austria (1), Finland (1)). One Hungarian sample was in cluster 5 (C5) and this sample was selected directly (RYEF_HUN18). Since 12 of the 32 cultivars originate from Hungary, it is not surprising that there is at least one Hungarian sample in each cluster. Interestingly, the 6 Canadian samples were all in C1, because they showed similar protein distributions, especially the highest amount of ALGL from 48 to 52% and the lowest amounts of ω-secalins (7.5–10%) and γ-75k-secalins (18–20%). For the final selection (Table 1), the focus lay on the differences of the samples, the country of origin, the differences between the M_r_ distributions and availability. At least one sample was selected from each cluster, whereas two samples were selected from C1, because this cluster contained the highest number of cultivars. Since C1 contained all Canadian and both German cultivars, one was selected from each country (WHE_CAN17, DAN_GER19). Sample WIB_HUN17 was chosen from C2, because of the significant difference in the M_r_ distribution of the prolamin fraction compared to the other cultivars in C2 (Table S5). One Polish (DAT_POL20) cultivar was selected from C3 and two samples were selected from C4 (Austrian ELI_AUS20 and Finnish RET_FIN20). The reason for the selection of those samples within the clusters, respectively, was the diversity of geographical origins. Since the European Union is the leading region in terms of rye production, the sample set covers a high variability and is considered to be representative.

### 3.7 In-depth characterization of the seven selected cultivars

#### 3.7.1 Protein content and gluten composition

**Fig. 3:**
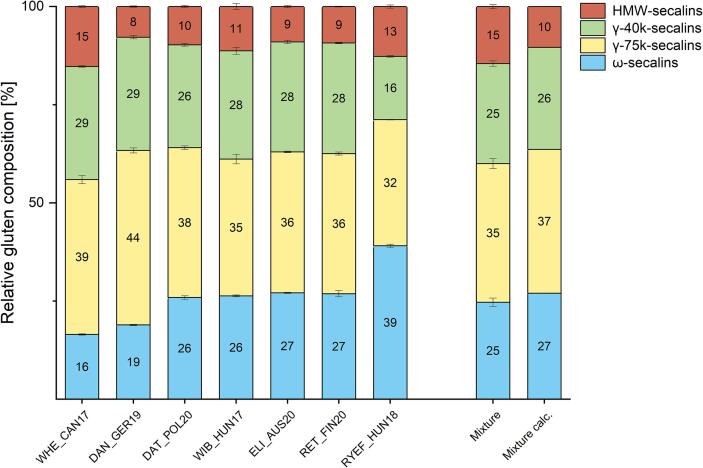
**Relative gluten composition of selected rye cultivars and their mixture**. The mixture consists of the flours of the selected 7 cultivars in equal proportions. Mixture calc. is the calculated composition resulting from the mean values. Error bars indicate the standard deviations (n = 3).

The protein content of the 7 selected cultivars measured with RP-HPLC ranged from 5.5 g/100 g (DAN_GER19) to 11.2 g/100 g (ELI_AUS20) (Table 1). The lowest and the highest gluten content was 3.0 g/100 g for WIB_HUN17 and 7.8 g/100 g for ELI_AUS20. The protein and gluten content corresponded well to the previously measured content of the 32 samples with 4.2 to 11.2 g/100 g of protein and 2.6 to 7.8 g/100 g of gluten (Table 1, Table S2). The proportion of ALGL ranged from 30% (ELI_AUS20) to 50.2% (WHE_CAN17) based on protein content. The relative gluten protein composition of the selected cultivars and the mixture is shown in Fig. 3. The average distribution of the seven samples was as follows: 37% γ-75k-secalins, 27% ω-secalins, 26% γ-40k-secalins and 10% HMW-secalins (Fig. 3, mixture calc.). The results obtained from lab-scale mixing of the selected flours in equal proportions (Fig. 3, mixture) showed a distribution of 35% γ-75k-secalins, 25% ω- secalins, 25% γ-40k- secalins and 15% HMW-secalins. The gluten composition of the mixture differed slightly from the calculated values, especially in the HMW-secalin proportion with 10% for the calculated mean and 15% for the flour mixture. This resulted in lower values for the other fractions compared to the calculation. One reason could be insufficient homogenization of comparatively small aliquots of the rye flours. Further work will use mixtures of grains prior to milling as reported before (Schall et al., 2020).

**Table 1:** Content of protein and gluten fractions of the selected rye cultivars measured by RP-HPLC.

Sample	Protein^a^	Gluten^b^	Prolamins	Glutelins	ALGL	ωs	γ-75k	γ-40k	HMW	PROL/GLUT ratio^c^
	[g/100 g]									[1]
RYEF_HUN18	8.62^C^	5.57^C^	4.70^C^	0.87^B^	3.04^B^	2.18^A^	1.79^C^	0.90^D^	0.71^A^	5.4
WIB_HUN17	5.80^E^	2.99^F^	2.40^F^	0.59^C^	2.81^C^	0.79^D^	1.04^G^	0.82^E^	0.34^C^	4.1
WHE_CAN17	6.14^D^	3.06^F^	2.49^F^	0.57^C^	3.08^B^	0.50^F^	1.21^D^	0.38^F^	0.47^B^	4.3
DAN_GER19	5.46^F^	3.60^E^	2.69^E^	0.91^B^	1.85^E^	0.68^E^	1.60^E^	1.04^C^	0.28^C^	3.0
ELI_AUS20	11.19^A^	7.83^A^	6.75^A^	1.08^A^	3.36^A^	2.12^A^	2.82^A^	2.19^A^	0.71^A^	6.3
DAT_POL20	6.40^D^	3.98^D^	3.35^D^	0.62^C^	2.42^D^	1.03^C^	1.52^F^	1.04^C^	0.39^BC^	5.4
RET_FIN20	10.39^B^	7.15^B^	6.10^B^	1.05^A^	3.24^AB^	1.92^B^	2.55^B^	2.01^B^	0.66^A^	5.8

a Sum of reduced prolamins, glutelins, albumins and globulins measured by RP-HPLC.

b Sum of reduced prolamins and glutelins measured by RP-HPLC.

c Ratio of reduced prolamins and glutelins measured by RP-HPLC. Rye protein fractions: ALGL: albumins and globulins, ωs: ω-secalins, HMW: high-molecular-weight secalins, γ-75k: γ-75k-secalins and γ-40k: γ-40k-secalins. The values are given as means (n = 3) and different capital letters indicate significant differences between the samples in each column (one-way ANOVA, Tukey’s post hoc test, p < 0.05).

Four samples (WIB_HUN17, ELI_AUS20, DAT_POL20 and RET_FIN20) showed similar protein distributions within and compared to the (calculated) mixture. The difference in the protein distribution and especially the high ω-secalin content of RYEF_HUN18 stood out compared to the other samples. WHE_CAN17 had the lowest ω-secalin (16%) and the highest HMW-secalin (15%) percentage. The sample DAN_GER19 showed the highest percentage for γ-75k-secalins (44%) and the lowest for HMW-secalins (8%). The γ-40k-secalins ranged from 16% (RYEF_HUN18) to 29% (WHE_CAN17 and DAN_GER19). For the selected samples the protein distribution fits mostly to the pattern discussed above and in past studies on rye proteins (Gellrich et al., 2003, Schalk et al., 2017, Rani et al., 2021). Four of the selected samples (WIB_HUN17, ELI_AUS20, DAT_POL20 and RET_FIN20) indicated an equal distribution between γ-40k-secalins and ω-secalins (±2%). The remaining three samples had unusual distributions with less γ-40k-secalins than ω-secalins in RYEF_HUN18, as well as comparatively low percentages of ω-secalins in WIB_HUN17 and DAN_GER19 (16% and 19%). These differences in protein distribution between the selected samples was one of the aims of this study to comprehensively cover the variability in rye gluten composition.

#### 3.7.2 SDS-PAGE

**Fig. 4:**
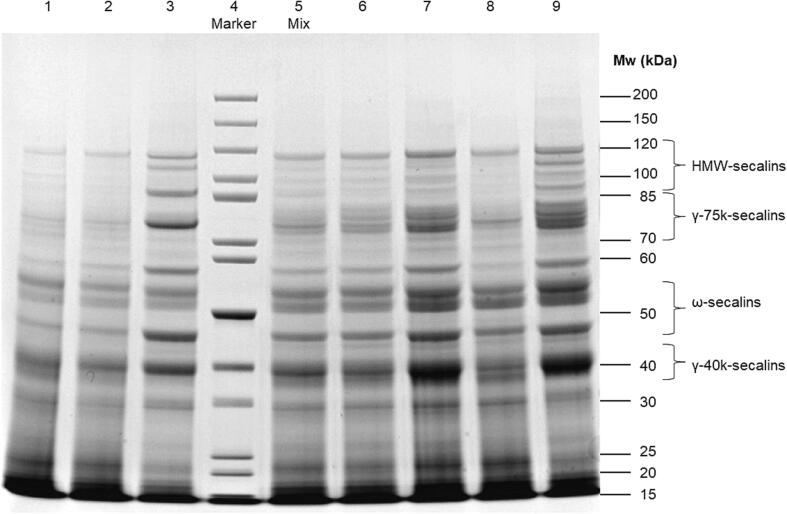
**SDS-PAGE of 7 selected rye flours and their mixture.** M: marker, 1: WHE_CAN17, 2: WIB_HUN17, 3: DAN_GER19, 4: molecular mass marker, 5: Mix: rye flour mixture, 6: DAT_POL20, 7: ELI_AUS20, 8: RYEF_HUN18, 9: RET_FIN20, Mw: molecular mass.

The M_r_ distribution of the rye flours and the mixture was further investigated by SDS-PAGE (Fig. 4). Based on previous studies, the secalins were divided according to their M_r_ (Shewry, 1982, Gellrich et al., 2003, Schalk et al., 2017). The bands specific for secalins are found between 85 and 120 kDa for HMW-secalins, 70–85 kDa for γ-75k-secalins, 45–55 kDa for ω-secalins and 35–45 for γ-40k-secalins. Gellrich et al. showed that the bands with lower M_r_ (<30 kDa) as well as bands in higher M_r_ ranges belong to the albumins and globulins. The bands of ELI_AUS20 and RET_FIN20 were relatively more pronounced, which could be due their high protein content (Table 1). The most outstanding sample was DAN_GER19, which has two strong bands in the area of the HMW-secalins. Furthermore, the band between 70 and 85 kDa was relatively pronounced compared to the other samples. This indicates a high content of HMW- and γ-75k secalins, corresponding very well to the results obtained with HPLC (Fig. 3, Table 1). The unusually high content of ω-secalins in RYEF_HUN18 compared to γ-40k- and γ-75k-secalins can be seen in the relatively more intense bands in the ω-secalin region.

## 4 Conclusion

This study is the first to report an in-depth characterization of the protein composition of 32 rye cultivars from various countries. For most of the samples the characterization showed a protein distribution pattern of rye gluten fractions as follows: γ-75k-secalins > γ-40k-secalins > ω-secalins > HMW-secalins. Hierarchical clustering of all quantitative data was used to select 7 representative cultivars from different countries (Austria, Canada, Finland, Germany, Hungary (2), Poland) with high variability in protein composition that are suitable as RM. Detailed analysis of the 7 selected cultivars and their blend supported our hypothesis that a mixture of different rye cultivars is more suitable compared to a single cultivar to reduce variability and be representative. The production of rye RM on a larger scale and its suitability for calibration of ELISAs will be addressed in future studies.

Furthermore, the average prolamin/glutelin ratio was 4.4 for rye and this translates to an estimated conversion factor from rye prolamins to gluten of 1.2, instead of 2. Consequently, the method used in ELISAs to calculate gluten content is inappropriate for rye and should be revised to reduce the current overestimation of rye gluten. Our results reinforce the need for more suitable RM for gluten quantitation and for further fundamental investigations on rye gluten proteins. These insights and further work on barley RM will improve gluten analytical methods in order to ensure food safety for celiac disease patients.

## Funding

Parts of the research reported here are related to the BME-EGA-02 - TKP2021 project supported by National Research, Development, and Innovation Fund of Hungary. Funded by the European Union (ERC, GLUTENOMICS, 101040437). Views and opinions expressed are however those of the author(s) only and do not necessarily reflect those of the European Union or the European Research Council Executive Agency. Neither the European Union nor the granting authority can be held responsible for them.

## CRediT authorship contribution statement

**Majlinda Xhaferaj:** Conceptualization, Data curation, Formal analysis, Investigation, Methodology, Writing – original draft. **Gabriella Muskovics:** Data curation, Formal analysis, Investigation, Methodology, Writing – review & editing. **Eszter Schall:** Investigation, Methodology, Writing – review & editing. **Zsuzsanna Bugyi:** Conceptualization, Funding acquisition, Project administration, Supervision, Writing – review & editing. **Sándor Tömösközi:** Conceptualization, Funding acquisition, Project administration, Supervision, Writing – review & editing. **Katharina A. Scherf:** Conceptualization, Funding acquisition, Project administration, Supervision, Writing – review & editing.

## Declaration of Competing Interest

The authors declare that they have no known competing financial interests or personal relationships that could have appeared to influence the work reported in this paper.

## Data Availability

Data will be made available on request.
