# Supplementary material for: Characterization of rye flours and their potential as reference material for gluten analysis
Source: Food Chem. 2023 May 15;408:135148. doi: 10.1016/j.foodchem.2022.135148 (PMC9875308; doi:10.1016/j.foodchem.2022.135148)
Supplement: Supplementary data 1 [file mmc1.pdf]

Characterization of rye flours and their potential as reference material for gluten analysis

Authors

Majlinda Xhaferaj[1], Gabriella Muskovics[2], Eszter Schall[2], Zsuzsanna Bugyi[2], Sándor Tömösközi[2], Katharina A. Scherf[1]

Affiliations

[1] Karlsruhe Institute of Technology, Institute of Applied Biosciences, Department of Bioactive and Functional Food Chemistry, Karlsruhe, Germany

[2] Budapest University of Technology and Economics, Department of Applied Biotechnology and Food Science, Research Group of Cereal Science and Food Quality, Budapest, Hungary

SUPPLEMENTARY MATERIAL

Table S1. *General information on the 32 samples.*

Sample name	Cluster Nr.	Sample code	Geographical origin	Year of collection	Provider
Hancock	1	HAN_CAN17	Canada	2017	Carrington Research Extension Center NDSU
Spooner	1	SPO_CAN17	Canada	2017	Carrington Research Extension Center NDSU
Rymin	1	RYM_CAN17	Canada	2017	Carrington Research Extension Center NDSU
Dacold	1	DAC_CAN17	Canada	2017	Carrington Research Extension Center NDSU
Aroostock	1	ARO_CAN17	Canada	2017	Carrington Research Extension Center NDSU
Hazlet	1	HAZ_CAN17	Canada	2017	Carrington Research Extension Center NDSU
Wheeler	1	WHE_CAN17	Canada	2017	Carrington Research Extension Center NDSU
Sangaste	1	SAN_EST19	Estonia	2019	Estonian Crop Research Institute
Vambo	1	VAM_EST19	Estonia	2019	Estonian Crop Research Institute
Daniello	1	DAN_GER19	Germany	2019	KWS Lochow GmbH
Performer	1	PER_GER19	Germany	2019	Saaten-Union GmbH
Dankowskie-Diament	1	DAD_HUN18	Hungary	2018	ELKH-ATK
Kaupo	1	KAU_LAT20	Latvia	2020	Institute of Agricultural Resources and Economics
Dankowskie-Granat	1	DAG_POL20	Poland	2020	DANKO Hodowla Roślin Sp. z o.o
Dankowskie-Agat	2	DA_HUN17	Hungary	2017	ELKH-ATK
Dankowskie-Diament	2	DAD_HUN17	Hungary	2017	ELKH-ATK
Wibro	2	WIB_HUN17	Hungary	2017	Galga-Agrár Kft
Dankowskie-Rubin	2	DR_HUN19	Hungary	2019	ELKH-ATK
Elego	3	ELEG_AUS20	Austria	2020	Saatzucht Edelhof
Elect	3	ELE_AUS20	Austria	2020	Saatzucht Edelhof
Elvi	3	ELV_EST19	Estonia	2019	Estonian Crop Research Institute
Dankowskie-Diament	3	DAD_HUN19	Hungary	2019	ELKH-ATK
Dankowskie-Skand	3	DAN_HUN19	Hungary	2019	ELKH-ATK
Dankowskie-Rubin	3	DAR_POL20	Poland	2020	DANKO Hodowla Roślin Sp. z o.o
Dankowskie-Turkus	3	DAT_POL20	Poland	2020	DANKO Hodowla Roślin Sp. z o.o
Elias	4	ELI_AUS20	Austria	2020	Saatzucht Edelhof
Rettaa	4	RET_FIN20	Finland	2020	Boreal Plant Breeding Ltd.
Dankowskie-Turkus	4	DANT_HUN19	Hungary	2019	ELKH-ATK
Rye Food	4	RYEF_HUN19	Hungary	2019	Kruppa Mag
Wibro	4	WIB_HUN18	Hungary	2018	Galga-Agrár Kft
Wibro	4	WIB_HUN19	Hungary	2019	Galga-Agrár Kft
Rye Food	5	RYEF_HUN18	Hungary	2018	Kruppa Mag

Table S2. *Summary of the analytical characterization of 32 rye flours given as range and mean (n = 3) ± standard deviation (SD).*

[g/100 g flour]	Parameters	Range			Mean ± SD		
		min		max			
	Moisture	8.17	-	13.02	10.36	±	1.04
	Fat	0.04	-	2.07	1.23	±	0.44
**Protein content**	Crude protein (N (Dumas) × 5.83)	5.24	-	13.19	9.09	±	1.67
	Protein (RP-HPLC)	4.21	-	11.19	7.21	±	1.60
**Gluten content**	Gluten (RP-HPLC)	2.57	-	7.83	4.33	±	1.32
	Gluten (GP-HPLC)	2.67	-	7.34	4.47	±	1.17
	Gluten (R5 ELISA)	2.34	-	59.12	23.06	±	13.71
	Gluten (G12 ELISA)	3.24	-	68.03	32.75	±	14.85
**Osborne fractions**	Prolamins (RP-HPLC)	1.96	-	6.71	3.53	±	1.18
	Prolamins red. (RP-HPLC)	2.06	-	6.75	3.54	±	1.17
	Prolamins (GP-HPLC)	2.23	-	6.89	3.89	±	1.24
	Prolamins red. (GP-HPLC)	2.21	-	6.38	3.69	±	1.05
	Glutelins (RP-HPLC)	0.51	-	1.15	0.79	±	0.18
	Glutelins (GP-HPLC)	0.46	-	1.17	0.78	±	0.17
	Prolamin/glutelin ratio[a]	2.97	-	6.26	4.44	±	0.80
**Protein fractions**	ALGL (RP-HPLC)	1.41	-	3.64	2.87	±	0.46
	ω-secalins (RP-HPLC)	0.44	-	2.34	1.04	±	0.50
	γ-75k-secalins (RP-HPLC)	1.03	-	2.82	1.63	±	0.43
	γ-40k-secalins (RP-HPLC)	0.74	-	2.19	1.20	±	0.39
	HMW-secalins (RP-HPLC)	0.20	-	0.71	0.45	±	0.12

[a]Ratio of reduced prolamins and glutelins measured by RP-HPLC

RP-HPLC: reversed-phase high-performance liquid chromatography; GP-HPLC: gel permeation HPLC; ALGL: albumins/globulins; HMW: high-molecular-weight; red: reduced with dithiothreitol

Table S3. *Moisture, protein and fat content as well as ELISA and GP-HPLC results. The values are given as means (n = 3). Different capital letters indicate significant differences between the samples in each column (one-way ANOVA, Tukey's post hoc test, $p < 0.05$).*

**Sample code**	**Moisture [%]**	**Protein (N × 5.83) [%]**	**Fat [%]**	**ELISA [g/100g]**		**GP-HPLC [g/100g]**			
				**Gluten R5**	**Gluten G12**	**Gluten[a]**	**Prolamins**	**Reduced Prolamins**	**Glutelins**
HAN_CAN17	9.5 ± 0.5	8.0 ± 0.2	1.1 ± 0.1	9.2 ± 2.0	30.7 ± 5.6	3.05^{MNO}	2.46^{OP}	2.52^{LMNOP}	0.53^{CD}
SPO_CAN17	9.6 ± 0.1	9.1 ± 0.1	0.8 ± 0.0	15.1 ± 2.2	27.8 ± 0.9	4.45^{FGH}	3.24^{KL}	3.44^{HIJK}	1.01^{A}
RYM_CAN17	9.5 ± 0.2	8.5 ± 0.0	0.7 ± 0.1	10.4 ± 1.3	38.8 ± 0.7	3.67^{JKLM}	2.92^{LM}	2.94^{JKLM}	0.73^{BC}
DAC_CAN17	9.6 ± 0.1	8.4 ± 0.1	1.0 ± 0.1	9.6 ± 3.9	30.4 ± 3.3	3.65^{JKLM}	2.83^{MN}	2.74^{LMNO}	0.91^{AB}
ARO_CAN17	9.5 ± 0.3	7.8 ± 0.1	0.9 ± 0.2	10.5 ± 3.1	28.2 ± 1.1	3.49^{KLMN}	2.45^{OP}	2.50^{MNOP}	0.99^{A}
HAZ_CAN17	9.5 ± 0.3	10.4 ± 0.1	1.7 ± 0.2	13.0 ± 2.1	31.1 ± 2.0	4.94^{EFG}	4.21^{GH}	4.06^{EFG}	0.88^{AB}
WHE_CAN17	9.3 ± 0.6	8.3 ± 0.1	1.9 ± 0.0	19.3 ± 0.9	20.6 ± 1.2	3.40^{KLMN}	2.60^{MNO}	2.61^{LMNO}	0.79^{AB}
SAN_EST19	9.9 ± 0.3	6.9 ± 0.0	1.1 ± 0.0	15.5 ± 1.5	19.0 ± 0.4	2.67^{O}	2.23^{P}	2.21^{OP}	0.46^{D}
VAM_EST19	10.4 ± 0.4	7.9 ± 0.1	0.9 ± 0.1	22.3 ± 0.7	27.4 ± 1.3	3.39^{KLMN}	3.37^{JK}	2.89^{KLMN}	0.50^{CD}
DAN_GER19	10.4 ± 0.3	7.2 ± 0.0	0.3 ± 0.1	10.5 ± 2.3	26.8 ± 1.7	3.97^{HIJK}	3.20^{KL}	3.10^{IJKL}	0.88^{AB}
PER_GER19	12.8 ± 0.0	5.2 ± 0.2	0.0 ± 0.1	10.9 ± 3.1	27.8 ± 1.5	3.76^{IJKL}	2.69^{MNO}	2.75^{LMNO}	1.01^{A}
DAD_HUN18	9.9 ± 0.4	7.2 ± 0.0	2.0 ± 0.3	23.6 ± 1.0	29.6 ± 1.0	2.86^{NO}	2.44^{OP}	2.33^{NOP}	0.53^{CD}
KAU_LAT20	10.0 ± 0.1	8.7 ± 0.0	1.4 ± 0.1	20.6 ± 0.4	26.7 ± 1.0	4.60^{FGH}	4.02^{H}	3.81^{EFGH}	0.79^{AB}
DAG_POL20	9.8 ± 0.3	8.1 ± 0.1	1.3 ± 0.3	21.4 ± 0.6	28.6 ± 1.8	4.42^{FGH}	3.62^{IJ}	3.43^{HIJK}	0.99^{A}
DA_HUN17	10.9 ± 0.1	7.9 ± 0.1	1.2 ± 0.1	8.9 ± 0.5	3.2 ± 0.1	3.54^{JKLM}	2.69^{MNO}	2.82^{LMN}	0.72^{BC}
DAD_HUN17	11.2 ± 0.1	8.1 ± 0.1	1.0 ± 0.0	9.8 ± 0.3	10.6 ± 1.1	3.19^{LMNO}	2.58^{MNOP}	2.47^{MNOP}	0.73^{BC}
WIB_HUN17	13.0 ± 0.1	7.5 ± 0.1	1.3 ± 0.3	2.3 ± 0.4	9.3 ± 0.3	3.07^{MNO}	2.49^{NOP}	2.60^{LMNOP}	0.46^{D}
DR_HUN19	10.3 ± 0.1	10.0 ± 0.0	1.3 ± 0.0	5.6 ± 1.2	15.9 ± 1.0	5.00^{DEF}	4.67^{EF}	4.28^{DEF}	0.72^{BC}
ELEG_AUS20	9.8 ± 0.4	11.1 ± 0.0	1.1 ± 0.2	31.9 ± 1.5	34.7 ± 0.4	6.05^{B}	5.75^{C}	5.52^{B}	0.53^{CD}
ELE_AUS20	10.2 ± 0.2	10.1 ± 0.1	1.2 ± 0.2	30.2 ± 0.7	38.8 ± 1.3	5.73^{BC}	4.92^{E}	4.82^{CD}	0.91^{AB}
ELV_EST19	10.9 ± 0.2	8.9 ± 0.1	1.1 ± 0.1	24.0 ± 0.9	40.2 ± 1.1	4.55^{FGH}	5.79^{C}	3.72^{FGH}	0.83^{AB}
DAD_HUN19	10.0 ± 0.1	9.9 ± 0.1	2.1 ± 0.0	37.0 ± 1.9	37.8 ± 0.5	4.95^{DEFG}	4.24^{GH}	4.04^{EFG}	0.91^{AB}
DAN_HUN19	10.3 ± 0.1	9.8 ± 0.1	1.2 ± 0.0	37.4 ± 0.7	38.7 ± 1.2	4.78^{EFG}	4.11^{GH}	3.99^{EFGH}	0.79^{AB}
DAR_POL20	11.6 ± 0.1	8.3 ± 0.1	1.2 ± 0.2	30.2 ± 1.8	42.1 ± 0.8	4.36^{GHI}	3.47^{JK}	3.52^{GHIJ}	0.84^{AB}
DAT_POL20	11.8 ± 0.1	10.8 ± 0.2	1.4 ± 0.1	30.8 ± 1.5	41.2 ± 1.3	4.15^{HIJ}	3.93^{HI}	3.69^{GHI}	0.46^{D}
ELI_AUS20	11.2 ± 0.3	13.2 ± 0.1	1.8 ± 0.0	40.6 ± 1.2	51.7 ± 0.9	7.11^{A}	6.89^{A}	6.38^{A}	0.73^{BC}
RET_FIN20	9.0 ± 0.1	12.4 ± 0.0	1.4 ± 0.4	39.9 ± 1.8	48.0 ± 2.0	6.72^{A}	6.46^{B}	5.87^{AB}	0.84^{AB}
DANT_HUN19	11.1 ± 0.4	9.0 ± 0.0	1.1 ± 0.0	42.8 ± 2.7	41.4 ± 0.3	4.86^{EFG}	4.19^{GH}	4.08^{EFG}	0.79^{AB}
RYEF_HUN19	8.2 ± 0.5	11.0 ± 0.1	1.7 ± 0.0	28.8 ± 2.3	56.6 ± 0.7	5.69^{BC}	4.89^{E}	4.70^{CD}	0.99^{A}
WIB_HUN18	11.7 ± 0.0	11.2 ± 0.1	1.2 ± 0.0	47.5 ± 0.3	49.2 ± 1.8	5.75^{BC}	5.35^{D}	4.88^{C}	0.88^{AB}
WIB_HUN19	9.4 ± 0.5	10.2 ± 0.2	1.3 ± 0.0	33.2 ± 1.7	56.6 ± 1.2	5.38^{CDE}	4.43^{FG}	4.37^{CDE}	1.01^{A}
RYEF_HUN18	10.7 ± 0.4	10.8 ± 0.1	1.9 ± 0.0	59.1 ± 3.2	68.0 ± 2.1	5.58^{BCD}	4.89^{E}	4.79^{CD}	0.79^{AB}

Table S4. *Protein composition of the 32 rye cultivars determined by RP-HPLC. Gluten: Sum of reduced prolamins and glutelins. Protein: Sum of reduced prolamins glutelins. albumins and globulins (ALGL). Values are given as means (n = 3) and different capital letters indicate significant differences between the samples in each column (one-way ANOVA, Tukey's post hoc test, p < 0.05).*

Sample code	PROL/GLUT Ratio	Protein	Gluten	Prolamins	Prolamins reduced	Glutelins	ALGL	ω-secalins	γ-75k-secalins	γ-40k-secalins	HMW-secalins
	[1]					[g/100g]					
HAN_CAN17	4.1	6.00^{PQ}	2.88^{RS}	2.21^{PQ}	2.32^{VW}	0.56^{MN}	3.12^{CDE}	0.47^{ST}	1.60^{LMN}	0.38^{P}	0.43^{EFGH}
SPO_CAN17	4.2	6.84^{KL}	3.69^{M}	2.94^{L}	2.98^{PQ}	0.71^{IJKL}	3.15^{BCDE}	0.68^{OP}	2.11^{D}	0.48^{O}	0.42^{EFGH}
RYM_CAN17	4.3	6.61^{LM}	3.41^{NO}	2.64^{M}	2.77^{QR}	0.65^{JKLM}	3.20^{BCD}	0.58^{QR}	1.89^{EFGH}	0.43^{OP}	0.51^{DEF}
DAC_CAN17	4.5	6.29^{NO}	3.18^{OPQ}	2.57^{MN}	2.60^{RST}	0.58^{MN}	3.11^{CDE}	0.60^{PQ}	1.78^{IJ}	0.38^{P}	0.26^{KL}
ARO_CAN17	4.1	5.80^{QR}	2.77^{ST}	2.14^{QR}	2.23^{WX}	0.55^{MN}	3.03^{DEF}	0.44^{T}	1.53^{MNO}	0.38^{P}	0.42^{EFGH}
HAZ_CAN17	4.3	7.99^{H}	4.75^{I}	3.78^{I}	3.86^{IJ}	0.90^{EFG}	3.23^{BCD}	0.93^{IJK}	2.73^{A}	0.61^{N}	0.48^{DEFG}
WHE_CAN17	4.3	6.14^{NOP}	3.06^{PQR}	2.35^{OPQ}	2.49^{STUV}	0.57^{MN}	3.08^{DE}	0.50^{QRST}	1.71^{JK}	0.38^{P}	0.47^{DEFG}
SAN_EST19	4.0	4.75^{T}	2.57^{T}	1.96^{R}	2.06^{X}	0.51^{N}	2.18^{K}	0.53^{QRS}	1.03^{R}	0.74^{M}	0.27^{JKL}
VAM_EST19	5.1	6.17^{NOP}	3.74^{LM}	3.07^{L}	3.13^{OP}	0.61^{LMN}	2.43^{J}	0.89^{JKL}	1.49^{NO}	0.98^{HI}	0.39^{GHIJ}
DAN_GER19	3.0	5.46^{S}	3.60^{MN}	2.63^{M}	2.69^{RS}	0.91^{DEFG}	1.85^{L}	0.68^{OP}	1.60^{KLM}	1.04^{GH}	0.28^{IJKL}
PER_GER19	3.9	4.21^{U}	2.79^{ST}	2.20^{PQ}	2.22^{WX}	0.57^{MN}	1.41^{M}	0.49^{RST}	1.17^{PQ}	0.94^{IJ}	0.20^{L}
DAD_HUN18	3.9	5.59^{RS}	2.97^{QRS}	2.40^{NOP}	2.37^{UVW}	0.61^{LMN}	2.62^{IJ}	0.70^{NO}	1.13^{QR}	0.75^{M}	0.99^{A}
KAU_LAT20	3.5	7.02^{JK}	4.57^{I}	3.64^{IJ}	3.55^{LMN}	1.02^{BCD}	2.45^{J}	1.00^{HI}	1.86^{FGHI}	1.28^{E}	0.43^{EFGH}
DAG_POL20	4.2	6.61^{LM}	3.82^{LM}	3.10^{L}	3.08^{P}	0.74^{IJK}	2.79^{GHI}	0.86^{KLM}	1.48^{O}	1.08^{FG}	0.40^{FGH}
DA_HUN17	3.4	6.08^{OPQ}	3.33^{O}	2.66^{M}	2.58^{RSTU}	0.75^{IJ}	2.75^{GHI}	0.83^{LM}	1.25^{P}	0.87^{KL}	0.38^{GHIJK}
DAD_HUN17	3.3	5.98^{PQ}	3.25^{OP}	2.52^{MNO}	2.50^{STUV}	0.76^{HIJ}	2.73^{HI}	0.70^{NO}	1.25^{P}	0.90^{JK}	0.40^{FGHI}
WIB_HUN17	4.1	5.80^{QR}	2.99^{QRS}	2.53^{MNO}	2.40^{TUVW}	0.59^{MN}	2.81^{FGHI}	0.79^{MN}	1.04^{R}	0.82^{L}	0.34^{HIJK}
DR_HUN19	3.7	8.55^{EF}	5.33^{FG}	4.20^{GH}	4.17^{GH}	1.15^{A}	3.23^{BCD}	1.27^{E}	1.95^{EF}	1.67^{C}	0.44^{EFGH}
ELEG_AUS20	5.8	9.34^{C}	6.28^{C}	5.35^{C}	5.36^{C}	0.92^{CDEF}	3.05^{DE}	1.45^{D}	2.32^{C}	2.04^{B}	0.34^{HIJK}
ELE_AUS20	5.6	8.22^{GH}	5.27^{G}	4.53^{F}	4.47^{F}	0.80^{GHI}	2.96^{EFG}	1.26^{E}	1.82^{GHI}	1.65^{C}	0.53^{DE}
ELV_EST19	4.9	6.76^{KL}	4.27^{J}	3.51^{JK}	3.55^{KLM}	0.72^{IJKL}	2.48^{J}	0.96^{HIJ}	1.70^{JKL}	1.24^{E}	0.37^{GHIJK}
DAD_HUN19	4.2	8.32^{FG}	5.01^{H}	4.02^{H}	4.04^{HI}	0.97^{BCDE}	3.31^{BC}	1.16^{F}	1.90^{EFG}	1.41^{D}	0.53^{DE}
DAN_HUN19	4.5	7.50^{I}	4.55^{I}	3.75^{I}	3.72^{JKL}	0.83^{FGHI}	2.95^{EFGH}	1.12^{FG}	1.76^{IJ}	1.27^{E}	0.40^{FGHI}
DAR_POL20	3.6	7.15^{J}	4.23^{JK}	3.32^{K}	3.32^{NO}	0.90^{DEFG}	2.93^{EFGH}	1.01^{HI}	1.61^{KLM}	1.13^{F}	0.47^{DEFG}
DAT_POL20	5.4	6.40^{MN}	3.98^{KL}	3.33^{K}	3.35^{MNO}	0.62^{KLMN}	2.42^{J}	1.03^{GH}	1.52^{MNO}	1.04^{GH}	0.39^{GHIJ}
ELI_AUS20	6.3	11.19^{A}	7.83^{A}	6.71^{A}	6.75^{A}	1.08^{AB}	3.36^{B}	2.12^{B}	2.82^{A}	2.19^{A}	0.71^{B}
RET_EST20	5.8	10.39^{B}	7.15^{B}	6.15^{B}	6.10^{B}	1.05^{AB}	3.24^{BCD}	1.92^{C}	2.55^{B}	2.01^{B}	0.66^{BC}
DANT_HUN19	4.6	7.64^{I}	4.60^{I}	3.81^{I}	3.78^{JK}	0.82^{FGHI}	3.04^{DE}	1.20^{EF}	1.79^{HIJ}	1.21^{E}	0.32^{HIJK}
RYEF_HUN19	4.5	9.39^{C}	5.75^{E}	4.69^{EF}	4.71^{E}	1.04^{ABC}	3.64^{A}	1.42^{D}	2.13^{D}	1.62^{C}	0.58^{CD}
WIB_HUN18	5.6	9.04^{D}	6.01^{D}	5.10^{D}	5.09^{D}	0.92^{DEF}	3.03^{DEF}	2.34^{A}	1.98^{E}	1.00^{H}	0.68^{BC}
WIB_HUN19	4.0	8.73^{E}	5.38^{FG}	4.27^{G}	4.31^{FG}	1.07^{AB}	3.36^{B}	1.29^{E}	1.94^{EF}	1.61^{C}	0.54^{DE}
RYEF_HUN18	5.4	8.62^{E}	5.57^{EF}	4.77^{E}	4.70^{E}	0.87^{EFGH}	3.04^{DE}	2.18^{B}	1.79^{HIJ}	0.90^{JK}	0.71^{B}

Table S5. *The relative molecular mass distribution with GP-HPLC categorized according to the following molecular mass ranges: 1: >66 kDa; 2: 66-29 kDa; 3: 29-12.4 kDa; 4: <12.4 kDa. Values are given as means (n = 3) and different capital letters indicate significant differences between the samples in each column (one-way ANOVA, Tukey's post hoc test, p < 0.05).*

**Sample code**	**Prolamins [%]**				**Reduced Prolamins [%]**				**Glutelins [%]**			
	**1**	**2**	**3**	**4**	**1**	**2**	**3**	**4**	**1**	**2**	**3**	**4**
HAN_CAN17	30.2^{FGHI}	11.1^{DE}	22.2^{N}	36.6^{EFGH}	5.4^{CDEF}	7.0^{QR}	53.3^{DEFGH}	34.3^{CDE}	9.8^{OP}	9.6^{M}	31.7^{I}	48.9^{AB}
SPO_CAN17	27.9^{LMN}	11.0^{EF}	22.8^{LMN}	38.3^{D}	5.1^{DEFGH}	7.4^{PQR}	54.9^{ABCD}	32.7^{DEF}	11.1^{IJKLMNO}	10.9^{IJK}	32.8^{FGHI}	45.2^{CD}
RYM_CAN17	28.1^{KLMN}	10.9^{EF}	22.8^{LMN}	38.1^{D}	5.5^{CDEF}	7.7^{NOPQR}	54.2^{BCDE}	32.6^{DEF}	10.8^{JKLMNOP}	11.5^{FGHI}	33.7^{EFGH}	44.0^{DE}
DAC_CAN17	28.5^{JKL}	11.0^{EF}	23.3^{KLM}	37.2^{DEFG}	3.8^{IJKL}	6.8^{R}	55.4^{ABC}	34.0^{DEF}	9.8^{MNOP}	9.5^{M}	31.4^{IJ}	49.2^{AB}
ARO_CAN17	31.3^{EF}	11.7^{B}	22.6^{MN}	34.4^{I}	5.6^{CDEF}	7.7^{NOPQR}	54.0^{BCDEF}	32.7^{DEF}	10.1^{LMNOP}	10.0^{KLM}	32.6^{GHI}	47.2^{BC}
HAZ_CAN17	27.7^{LMNO}	10.6^{GHI}	23.7^{JK}	37.9^{D}	5.3^{CDEF}	7.6^{OPQR}	55.1^{ABCD}	32.0^{FG}	12.7^{GH}	11.6^{FGHI}	34.6^{CDEF}	41.1^{FGH}
WHE_CAN17	30.9^{FGH}	10.8^{EFG}	22.1^{N}	36.2^{FGH}	5.1^{DEFGH}	7.4^{PQR}	54.0^{BCDEF}	33.5^{DEF}	9.5^{P}	9.7^{M}	31.4^{IJ}	49.3$^{A B}$
SAN_EST19	30.3^{FGHI}	11.5^{BC}	21.0^{O}	37.2^{DEFG}	5.7^{CDE}	8.2^{MNOP}	51.4^{HIJKL}	34.7^{BCD}	11.6^{HIJKL}	10.9^{IJK}	34.0^{DEFGH}	43.6^{DEF}
VAM_EST19	29.3^{IJKO}	11.0^{EF}	23.9^{JK}	35.9^{GH}	5.5^{CDEF}	8.1^{MNOPQ}	50.1^{KLM}	36.2^{BC}	11.0^{IJKLMNOP}	10.8^{IJKL}	34.5^{DEFG}	43.8^{DE}
DAN_GER19	37.1^{A}	12.0^{A}	27.5^{EF}	23.4^{O}	4.2^{GHIJK}	12.8^{EF}	55.7^{AB}	27.3^{IJ}	15.0^{CDE}	18.2^{A}	36.7$^{A B}$	30.2^{N}
PER_GER19	33.8^{BC}	8.9^{M}	27.4^{EF}	29.9^{LM}	3.3^{KL}	9.7^{JKL}	54.0^{BCDEF}	33.0^{DEF}	16.0^{ABCD}	16.7^{B}	31.4^{IJ}	35.9^{KL}
DAD_HUN18	30.5^{FGHI}	11.0^{EF}	22.6^{MN}	35.9^{GH}	7.2^{B}	13.5^{E}	50.2^{JKLM}	29.1^{HI}	11.3^{HIJKLMN}	10.2^{KLM}	31.7^{I}	46.8^{BC}
KAU_LAT20	31.0^{FG}	9.1^{M}	29.2^{CD}	30.8^{L}	4.1^{HIJKL}	10.1^{IJK}	51.9^{GHIJK}	34.0^{DEF}	16.6^{AB}	16.6^{B}	32.7^{GHI}	34.1^{LM}
DAG_POL20	34.1^{BC}	10.6^{GHI}	28.0^{E}	27.4^{N}	4.7^{EFGHIJ}	11.5^{G}	52.0^{FGHIJK}	31.8^{FG}	15.6^{BCDE}	15.1^{CD}	31.4^{IJ}	37.9^{IJK}
DA_HUN17	27.3^{LMNO}	10.2^{JK}	22.6^{MN}	39.9^{C}	9.4^{A}	21.8^{AB}	47.0^{NO}	21.8^{K}	11.6^{GHIJK}	11.2^{HIJ}	32.3^{HI}	44.8^{CD}
DAD_HUN17	29.7^{HIJ}	10.2^{JK}	22.8^{LMN}	37.4^{DEF}	6.5^{BC}	11.4^{G}	53.2^{DEFGH}	28.9^{HI}	12.4^{GHI}	11.3^{GHIJ}	32.8^{FGHI}	43.5^{DEF}
WIB_HUN17	25.4^{Q}	10.5^{HI}	22.8^{LMN}	41.3^{B}	9.5^{A}	19.3^{C}	45.0^{O}	26.2^{J}	10.3^{KLMNOP}	9.8^{LM}	29.6^{JK}	50.3^{A}
DR_HUN19	26.6^{OPQ}	10.1^{JK}	23.5^{JKL}	39.7^{C}	8.8^{A}	22.1^{A}	49.8^{LM}	19.3^{L}	12.4^{GHI}	12.0^{EFGH}	33.6^{EFGH}	42.0^{EFG}
ELEG_AUS20	33.2^{CD}	10.0^{K}	28.8^{D}	28.0^{N}	6.0^{CD}	10.9^{GHI}	50.8^{IJKL}	32.3^{EF}	14.8^{DE}	15.4^{CD}	34.0^{DEFGH}	35.8^{KL}
ELE_AUS20	31.2^{EF}	11.1^{E}	26.8^{F}	30.9^{KL}	5.6^{CDEF}	9.6^{JKL}	46.0^{O}	38.8^{A}	14.4^{EF}	11.3^{HIJ}	28.4^{K}	45.9^{CD}
ELV_EST19	29.9^{GHI}	11.1^{E}	23.4^{KL}	35.6^{HI}	5.3^{DEFG}	8.5^{LMNOP}	54.3^{BCDE}	31.9^{FG}	11.1^{IJKLMNO}	12.0^{EFGH}	37.9^{A}	39.0^{HIJ}
DAD_HUN19	28.5^{JKL}	10.3^{IJK}	24.0^{IJK}	37.0^{DEFG}	7.4^{B}	15.7^{D}	53.9^{BCDEFG}	23.1^{K}	11.8^{GHIJ}	12.3^{EF}	35.4^{BCDE}	40.5^{GHI}
DAN_HUN19	27.6^{LMNO}	10.4^{IJ}	24.3^{IJ}	37.7^{DE}	5.2^{DEFGH}	8.7^{LMNO}	52.2^{EFGHIJ}	33.8^{DEF}	11.3^{HIJKLM}	11.7^{EFGHI}	36.4^{ABC}	40.6^{GH}
DAR_POL20	28.2^{KLM}	9.6^{L}	32.9^{A}	29.4^{M}	2.9^{L}	11.4^{GH}	56.8^{A}	28.8^{HI}	17.4^{A}	18.8^{A}	32.1^{HI}	31.7^{MN}
DAT_POL20	34.9^{B}	10.7^{FGH}	29.8^{BC}	24.6^{O}	5.0^{DEFGHI}	11.7^{FG}	53.4^{CDEFGH}	30.0^{GH}	16.4^{ABC}	14.5^{D}	29.8^{JK}	39.3^{HI}
ELI_AUS20	32.4^{DE}	10.6^{GHI}	29.6^{CD}	27.4^{N}	4.5^{FGHIJ}	10.2^{HIJK}	48.5^{MN}	36.8^{AB}	13.1^{FG}	14.7^{D}	35.6^{BCD}	36.7^{JKL}
RET_FIN20	19.9^{R}	10.4^{IJ}	15.6^{P}	54.2^{A}	3.6^{JKL}	10.8^{GHIJ}	52.3^{EFGHIJ}	33.3^{DEF}	15.7^{BCDE}	16.1^{BC}	33.1^{FGHI}	35.1^{L}
DANT_HUN19	28.0^{KLMN}	10.4^{IJ}	25.2^{GH}	36.4^{EFGH}	5.3^{CDEF}	8.8^{LMN}	52.5^{EFGHI}	33.3^{DEF}	11.4^{HIJKL}	12.2^{EFG}	36.8^{AB}	39.6^{GHI}
RYEF_HUN19	27.1^{MNO}	10.5^{HI}	24.8^{HI}	37.7^{DE}	9.0^{A}	20.9^{B}	51.6^{HIJKL}	18.5^{L}	12.1^{GHIJ}	12.5^{EF}	35.2^{BCDE}	40.2^{GHI}
WIB_HUN18	27.5^{LMNO}	10.6^{GHI}	29.8^{BC}	32.2^{JK}	5.7^{CDE}	8.4^{MNOP}	52.2^{EFGHIJ}	33.6^{DEF}	11.5^{HIJKL}	10.3^{JKLM}	34.3^{DEFG}	43.8^{DE}
WIB_HUN19	26.9^{NOP}	9.7^{L}	25.7^{G}	37.8^{DE}	5.7^{CDE}	9.3^{KLM}	55.7^{AB}	29.3^{HI}	11.9^{GHIJ}	12.7^{E}	35.0^{BCDE}	40.4^{GHI}
RYEF_HUN18	25.7^{PQ}	11.4^{CD}	30.4^{B}	32.5^{J}	7.4^{B}	16.2^{D}	50.6^{IJKL}	25.9^{J}	9.8^{NOP}	9.8^{LM}	35.4^{BCDE}	45.0^{CD}
